# Longitudinal Outcomes of Cumulative Impact Exposure on Oculomotor Functioning in Professional Motorsport Drivers

**DOI:** 10.1001/jamanetworkopen.2023.11086

**Published:** 2023-05-02

**Authors:** Danielle M. Ransom, Luis M. Ahumada, P. Patrick Mularoni, Terry R. Trammell

**Affiliations:** 1 Division of Neuropsychology, Johns Hopkins All Children’s Hospital, St Petersburg, Florida; 2 Institute for Brain Protection Sciences, Johns Hopkins All Children's Hospital, St Petersburg, Florida; 3 Institute for Clinical and Translational Research, Johns Hopkins All Children’s Hospital, St Petersburg, Florida; 4 Division of Sports Medicine, Johns Hopkins All Children's Hospital, St Petersburg, Florida; 5 Johns Hopkins University School of Medicine, Baltimore, Maryland; 6 Trammell Consulting LLC, Indianapolis, Indiana

## Abstract

**Importance:**

Professional motorsport drivers are regularly exposed to biomechanical forces comparable with those experienced by contact and collision sport athletes, and little is known about the potential short-term and long-term neurologic sequelae.

**Objective:**

To determine whether cumulative impact exposure is associated with oculomotor functioning in motorsport drivers from the INDYCAR professional open-wheel automobile racing series.

**Design, Setting, and Participants:**

This is a longitudinal retrospective cohort study conducted across 3 racing seasons (2017-2019). Statistical analyses were conducted in November 2021. Data were retrieved from a secondary care setting associated with the INDYCAR series. INDYCAR series drivers who participated in 3 professional level racing seasons and were involved in at least 1 contact incident (ie, crash) in 2 of the 3 seasons were included in the study.

**Exposure:**

Cumulative acceleration and deceleration forces and total contact incidents (ie, crashes) measured via accident data recorder third generation chassis and ear accelerometers.

**Main Outcomes and Measures:**

Postseries oculomotor performance, including predictive saccades, vergence smooth pursuit, and optokinetic nystagmus, was measured annually with a head-mounted, clinical eye tracking system (Neurolign Dx 100).

**Results:**

Thirteen drivers (mean [SD] age, 29.36 [7.82] years; all men) sustained median resultant acceleration forces of 38.15 *g* (observed range, 12.01-93.05 *g*; 95% CI, 30.62-65.81 *g*) across 81 crashes. A 2-way multivariate analysis of variance did not reveal a statistically significant association between ear and chassis average resultant *g* forces, total number of contact incidents, and racing season assessed (*F*_9,12_ = 0.955; *P* = .54; Wilks Λ = 0.44).

**Conclusions and Relevance:**

In this cohort study of professional drivers from the INDYCAR series, there were no statistically significant associations among cumulative impact exposure, racing season assessed, and oculomotor performance. Longitudinal studies across racing seasons using multidimensional examination modalities (eg, neurocognitive testing, advanced imaging, biomarkers, and physical examination) are critical to understand potential neurological and neurobehavioral sequelae and long-term consequences of cumulative impact exposure.

## Key Points

**Question:**

Is cumulative impact exposure associated with oculomotor performance in professional motorsport drivers?

**Findings:**

In this longitudinal cohort study of 13 INDYCAR series drivers, there were no statistically significant associations between cumulative impact exposure and oculomotor performance.

**Meaning:**

These findings reflect the relative stability of oculomotor performance despite exposure to the highest levels of cumulative impact conferred through multiple crashes over a 3-year period.

## Teaser

This longitudinal cohort study investigates whether cumulative impact exposure is associated with oculomotor functioning in motorsport drivers from the INDYCAR professional open-wheel automobile racing series.

## Introduction

Efforts are under way to characterize neurobehavioral sequelae and long-term consequences of impact-related acceleration and deceleration and rotational force exposure in athletes across all levels and sports.^1,2,3,4,5,6,7,8,9^ Motorsport is no exception, with recent public and professional concern shifting primary focus from severe traumatic brain injuries to mild traumatic brain injuries or concussion and subconcussive force exposure. This effort has been aided by medical and engineering innovations to improve driver safety.^10^ Compared with athletes engaged in contact and collision sports, motorsport drivers are exposed to high longitudinal and rotational forces as a function of event demands, even in the absence of a crash.^11,12,13^ Force exposure varies as a function of motorsport type, event class or series, terrain and race conditions (eg, oval track vs road), and/or crash event characteristics, including changes in velocity, peak acceleration, number of impacts, and vehicle dynamics.^14^ The combination of these factors results in a unique biomechanical force signature translated to drivers in a crash and is accumulated over time.^10^

Very few studies have examined the prevalence rate of concussion in professional motorsport. Limited data from the INDYCAR series from 1981 to 1984 indicated 13 reported concussions, defined as hospital admission for loss of consciousness.^15^ Now with nearly 40 years of data since prevalence rates were first published, these early data from 1981 to 1984 have restricted generalizability and do not reflect current diagnostic criteria or innovations in engineering technology. Advances in vehicle safety mandated by the Fédération Internationale de l’Automobile (FIA), including the head and neck support device introduced by the INDYCAR series in 2003, safety harnesses, airbags, and improvements to track safety such as the energy-absorbing steel and foam energy reduction barrier developed by the INDYCAR series in 2002, have substantially reduced force translation to the driver and decreased impact-related injuries across the severity spectrum.^16,17,18,19^ Despite significant improvements in driver safety and the frequency of reported concussions in recent years, drivers are regularly exposed to unique biomechanical forces vs contact and collision sport athletes, and little is known about the potential short-term and long-term consequences of this exposure.

In the event of suspected concussion, the most recent statement^3^ from the International Consensus Conference on Concussion in Sport emphasizes the importance of a multidimensional screening examination comprising symptom report, neurocognitive evaluation, physical examination, and vestibular assessment.^20^ Concussion protocols^20^ consistent with this international consensus statement^3^ have been adopted by all major governing sporting organizations, including the FIA. Leading the development and implementation of the motorsport concussion protocol, the INDYCAR series has developed policies and procedures for preseason baseline testing, trackside assessment, and return to racing. The INDYCAR protocol mandates that drivers are medically evaluated if head accelerations sustained during a contact incident exceed 80 *g*, with return to racing decisions informed by a range of objective and subjective measures comparing driver functioning to preseason baseline measures. Even with formalized procedures for concussion, concussion education, symptom recognition, and injury management, concussion symptoms are often underreported or unrecognized in athletes of all levels and across sports, including motorsport drivers, necessitating the use of objective tools to assess symptoms in the acute phase postinjury and to document cumulative exposure over the course of the racing season.^21,22,23,24^

Oculomotor assessment has been indicated as an objective measure of concussion as part of a multidimensional evaluation. ^25,26^ Afferent and efferent visual pathways are widely distributed throughout the brain and appear to be particularly vulnerable to disruptions in neurotransmission arising from diffuse shear injury associated with concussive and subconcussive impacts.^27,28^ Postinjury visual dysfunction may include abnormalities in accommodation and convergence, saccadic and/or pursuit eye movements, pupillary light reflex, extraocular motility, and stereoacuity and may contribute to blurred vision, eye fatigue, and difficulty focusing.^28,29^ Characterizing postinjury visual disturbance has proven to be a challenge due to measurement limitations of many available tools. Vestibular oculomotor screening for vestibular ocular dysfunction via eye movement tracking and associated symptom provocation has demonstrated validity in distinguishing patients with and without concussion and is increasingly used by health care professionals in baseline and postinjury testing.^30,31,32,33^ Oculomotor devices may additionally provide enhanced sensitivity and specificity in identifying acute and chronic impairment through enhanced measurement and recording technology.^34,35,36,37^ Despite the importance of visual functioning in balancing safety and performance in the high stakes motorsport environment, to our knowledge, no published studies have explored the utility of oculomotor tracking devices with professional motorsport drivers.

The purposes of this study were to (1) characterize cumulative impact exposure and (2) investigate the association between cumulative impact exposure and oculomotor functioning in a longitudinal cohort of professional INDYCAR series drivers. We hypothesized that greater impact exposure would be associated with less efficient oculomotor functioning over time.

## Methods

### Design and Participants

This cohort study was reviewed and approved by INDYCAR Medical and was determined to be exempt from institutional review board or ethics committee review. All drivers provided broad consent to the data collection, data analysis, and data use. The study adhered to the Strengthening the Reporting of Observational Studies in Epidemiology (STROBE) reporting guideline for cohort studies. This study used a longitudinal retrospective cohort design with data retrieved from a secondary care setting associated with the INDYCAR professional open-wheel automobile racing series. Participants included professional drivers who participated in 3 professional racing seasons and were involved in at least 1 contact incident (ie, crash) in 2 of the 3 seasons between 2017 and 2019.

### Impact Exposure Monitoring

All drivers in the INDYCAR series cohort were equipped with FIA homologated 8860 helmet from 2015 or newer as required by season-specific rules, a custom-fitted accelerometer placed in the ear canal with a 6-axis measurement, and a separate chassis accelerometer uniformly fixed to the floor of the chassis near the center of its mass. Each car in the series was outfitted with the same chassis and aerodynamic package. The INDYCAR series has used the Accident Data Recorder, third generation (ADR-3) data collection system for ear and chassis accelerometers since 2010. The ADR-3 was sourced from Delphi Motorsports and maintained in house by INDYCAR Safety Engineering. Events were recorded when sensors were triggered by a 5-*g* acceleration in any axis, storing 120 seconds of pretrigger data and 60 seconds of posttrigger data in the ADR-3 data log. The chassis sensor and separate right and left ear sensors recorded peak resultant acceleration and deceleration forces in 3 orthogonal axes (lateral, longitudinal, and vertical) at a sampling rate of 1000 Hz. Season-specific ADR-3 chassis and ear recordings and total contact incidents (ie, crashes) from practice sessions, qualifying events, and races were used as indicators of cumulative impact exposure. Contact incidents were monitored in real time by INDYCAR Race Control and confirmed by review of ADR-3 data, crash damage inspection, and video by INDYCAR Accident Investigation personnel.

### Oculomotor Performance

In preparation for the INDYCAR series annual opening each March, each driver in the INDYCAR series is required to undergo a preseason baseline physical and neurocognitive examination during scheduled dates in the preceding months (December or January). Since 2017, examination has included assessment of oculomotor functioning, vestibular functioning, and reaction time functioning using the Neurolign Dx 100 device, a portable, head-mounted display and eye-tracking system that is certified as a Nonsignificant Risk Device by the US Food and Drug Administration and approved for clinical use (21 CFR 882.1460). This device has demonstrated clinical utility in differentiating adults with concussion from healthy controls immediately after injury, with normalization of performance associated with symptom resolution and recovery in the subacute injury period.^38,39^ To our knowledge, no studies have been published exploring the utility of the Neurolign Dx 100 device in a motorsport population. For this study, oculomotor performance data was extracted from subtests administered as part of the Neurolign Dx 100 battery, including predictive saccades (horizontal time and displacement), vergence smooth pursuit (eye correlation inward and outward), and optokinetic nystagmus (percentage of gain asymmetry).

### Statistical Analysis

We first engaged in a detailed data examination of the distributions of ear and chassis accelerometer recordings. We then eliminated data points exceeding 2 SD from the mean to screen out potential noise at the higher end of the spectrum to avoid undue influence of statistical outliers on the results. To establish distinct grouping variables based on the distribution of extracted cumulative impact exposure, 2 cluster analyses were conducted to categorize (1) ear and chassis mean resultant acceleration or deceleration forces and (2) the total number of contact incidents. Finally, we conducted a 2-way multivariate analysis of variance to examine the association of cumulative impact exposure with changes in oculomotor performance over time. A total of 3 independent variables were included: the 2 grouping variables derived from the cluster analyses, the racing season assessed, and the interaction among the 3 variables. A total of 6 dependent variables were used to indicate changes in oculomotor performance, including relative differences on examination in 2018 and 2019 in comparison to 2017 baseline examination of predictive saccades, vergence smooth pursuit, and optokinetic nystagmus (oculomotor performance stratified by racing season is reported in the Table). The α was set at .05 using 2-sided *F* tests, and statistical analyses were conducted using R statistical software version 4.1.2 (R Project for Statistical Computing) and SPSS Statistics software version 27 (IBM). Statistical analyses were completed in November 2021.

**Table.** Driver Oculomotor Performance Measured with the Neurolign Dx 100 by Racing Season (Year)

Measure^a^	2017		2018		2019	
	Score, mean (SD)	Score, median (IQR)	Score, mean (SD)	Score, median (IQR)	Score, mean (SD)	Score, median (IQR)
Optokinetic nystagmus						
Left horizontal	−0.17 (2.78)	−0.16 (−1.94-1.95)	0.24 (3.94)	1.15 (0.94-1.46)	0.13 (3.58)	0.62 (−1.15-1.60)
Left vertical	0.06 (5.36)	0.24 (−3.91-2.59)	0.90 (5.35)	−0.25 (−2.09-2.59)	0.68 (6.33)	−0.07 (−2.70-5.30)
Right horizontal	0.29 (2.99)	0.39 (−2.50-1.63)	0.36 (2.42)	0.76 (−1.19-1.80)	−0.91 (4.07)	−0.70 (−2.19-1.79)
Right vertical	0.75 (5.41)	1.06 (−1.47-2.87)	1.19 (5.92)	−0.03 (−2.12-1.48)	−0.74 (5.43)	−1.39 (−3.82-1.95)
Predictive saccades						
Left horizontal	1.44 (2.66)	1.46 (−0.79-2.96)	1.47 (3.37)	2.22 (1.42-3.21)	1.90 (2.11)	2.50 (0.49-3.00)
Left vertical	2.16 (6.02)	3.80 (−2.90-6.89)	1.05 (4.67)	−0.16 (−2.22-0.64)	−0.61 (4.68)	−0.39 (−2.30-1.89)
Right horizontal	1.31 (2.50)	1.56 (−0.38-2.86)	1.10 (2.24)	1.99 (0.72-2.19)	0.45 (2.40)	0.73 (−0.33-0.82)
Right vertical	3.48 (7.02)	2.53 (0.04-5.75)	1.97 (5.28)	−0.13 (−0.86-1.57)	−1.90 (4.07)	−1.68 (-3.57-0.82)
Vergence pursuit						
Left horizontal	3.57 (14.35)	-0.31 (−2.55-1.43)	0.03 (3.45)	0.83 (0.29-1.37)	−0.25 (3.10)	0.19 (−1.54-1.95)
Left vertical	1.44 (6.02)	0.61 (−2.82-5.19)	1.50 (5.00)	0.15 (−1.85-2.38)	1.35 (5.70)	1.16 (−0.08-3.95)
Right horizontal	−3.70 (11.65)	-0.68 (−2.63-1.18)	−0.50 (2.28)	0.36 (−2.63-0.78)	−6.12 (16.07)	−1.01 (−4.30-0.15)
Right vertical	1.81 (7.27)	2.13 (−0.25-5.18)	2.08 (5.51)	0.23 (−1.24-1.67)	1.2 (7.87)	−0.74 (−1.44-3.42)

^a^ Oculomotor scores have been normalized to z scores (mean = 0.00, SD = −1.00)

## Results

The initial data extraction included 19 drivers who were involved in a contact incident in 2 of the 3 seasons included in this study (2017-2019). After outliers based on series participation and accelerometer recordings were removed, a cohort of 13 male drivers was included in final analyses (mean [SD] age, 29.36 [7.82] years; range, 21-42 years). Results indicated that drivers sustained median resultant acceleration forces of 38.15 *g* (observed range, 12.01-93.05 *g*; 95% CI, 30.62-65.81 *g*) across 81 crashes. Separate cluster analyses of cumulative impact exposure identified the following: (1) a 3-group solution for ear or chassis accelerometer recordings of mean resultant acceleration or deceleration forces (low cumulative impact, 8 drivers; mean [SD], 24.45 [6.88] *g*; medium cumulative impact, 4 drivers; mean [SD], 85.95 [9.85] *g*; high cumulative impact, 1 driver; 122.80 *g*) and (2) a 2-group solution for number of contact incidents (low, 5 incidents; mean [SD], 26.95 [24.42] *g*; high, 8 incidents; mean [SD], 86.60 [40.46] *g*). Cumulative impact exposure by driver is illustrated in the Figure.

**Figure.**
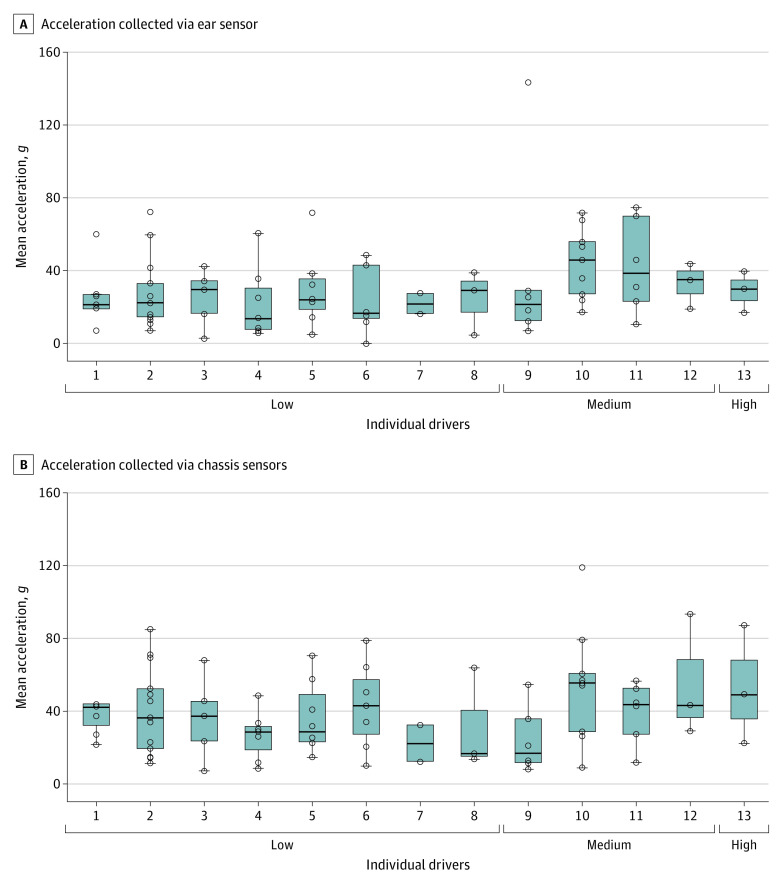
Acceleration Across Impacts in a Cohort of 13 INDYCAR Series Drivers, 2017-2019 Graphs show mean resultant acceleration collected via ear sensor (A) and via chassis sensor (B). Drivers are grouped according to cumulative impact exposure (low, medium, and high). Circles denote individual impact incidents, lines within boxes denote means, tops and bottoms of boxes denote SDs, and error bars denote 95% CIs.

A 2-way multivariate analysis of variance did not reveal a statistically significant interaction between ear or chassis mean resultant acceleration or deceleration forces, total number of contact incidents, and racing season assessed (*F*_9,12_ = 0.955; *P* = .54; Wilks Λ = 0.44). Simple analyses showed that no independent variables, including ear and chassis mean resultant acceleration and deceleration forces, total number of contact incidents, or racing season assessed, had statistically significant associations with changes in oculomotor performance in the 3-year study period.

## Discussion

This cohort study sought to characterize cumulative impact exposure and investigate the association with oculomotor functioning in INDYCAR series drivers. The 13 drivers included in the study were exposed to high acceleration and deceleration forces over time and were involved in a recorded crash in 2 of 3 racing seasons. We hypothesized that greater exposure would be associated with a relative decline in oculomotor functioning over time. However, the findings did not support our hypothesis, because we did not observe statistically significant associations of cumulative impact exposure or racing season assessed with oculomotor performance over time. These findings are consistent with studies^40,41,42,43^ in contact and collision sports that failed to find associations between exposure and short-term and long-term neurobehavioral sequelae.

There is growing concern and ongoing debate among the public and health care professionals regarding the safety of contact and collision sports for athletes across the life span. Numerous scientific efforts across the world are underway to better understand the potential short-term and long-term consequences of repetitive head trauma.^1,2,3,4,5,6,7,8,9^ Within motorsport, historical emphasis has been placed on reducing catastrophic injuries, with the study of concussion and subconcussive impact exposure lagging behind contact and collision sport counterparts.^10^ Innovations in vehicle and track engineering have led to greatly reduced injuries of all severity, such as the reduced frequency of reported concussions in the last decade in comparison to the 1980s, even with substantial changes in concussion diagnostic criteria. Little is known, however, about the impact of biomechanical force signatures unique to motorsport and the potential short-term and long-term consequences of cumulative force exposure in drivers. To our knowledge, this study is the first to explore neurological outcomes of cumulative exposure in a professional motorsport cohort.

The nature of motorsport relies on an exacting balance of speed and accuracy in an extremely high-stakes setting where any threat to an individual driver’s decision-making precision has the potential to increase crash risk and reduce safety for the entire driver field. Despite the robust protocol for monitoring driver health, drivers are frequently exposed to concussive and subconcussive forces with unknown potential for neurobehavioral sequelae and long-term consequences. Furthermore, surveyed motorsport drivers and medical personnel across professional series within the FIA have demonstrated gaps in concussion-related knowledge and disparities in injury-reporting attitudes.^44^ The high stakes inherent to motorsport coupled with limited knowledge and inconsistent injury reporting have the potential to undermine the engineering and medical advances from the last decade that contributed to improved driver safety and reduced injury risk.

Like other contact and collision sports, a multidimensional toolkit composed of subjective symptom reports and objective measures is necessary to the team health care practitioner’s examination at baseline and in the acute, subacute, and chronic stages of impact exposure. Assessment of oculomotor functioning may serve as an objective component of the examination as an important proxy for aspects of neurological functioning known to be impacted by brain injury. Although oculomotor examination has been used by the INDYCAR series only since 2017, it is a logical extension that reduced cumulative impact exposure associated with engineering innovations has contributed to overall improvements to driver safety over time, including relatively preserved oculomotor functioning.

### Clinical Implications

Given the high stakes associated with professional motorsport competition and the important role of oculomotor functioning in driver safety, objective evaluation with serial monitoring should remain a priority in the multidimensional examination of driver health. For drivers involved in crashes exceeding the specified gravitational force threshold, evaluation with reliable and valid multidimensional tools (eg, the Sport Concussion Assessment Tool, Fifth Edition^45^) is critical to reduce the potential for catastrophic consequences and to prioritize driver safety for the entire racing field. Oculomotor examination may serve as an additional objective measure of neurological functioning sensitive to disruptions in neurotransmission in the event of an acute crash and could also be used when monitoring the cumulative impact of acceleration and deceleration force exposure over time. Just as engineering advances within the INDYCAR series and across the FIA have contributed to fewer catastrophic injuries, multidimensional examination of driver health in the acute, subacute, and long-term time points are critical components to maintaining driver safety.

### Limitations

Although the current study has several methodological strengths and is the first, to our knowledge, to explore sequelae associated with impact exposure in INDYCAR series drivers, it does have limitations. First, this study used longitudinal assessment of oculomotor functioning as the primary outcome. Focus on any 1 domain in the assessment of brain health will substantially limit sensitivity to the full range of potential sequelae associated with acute or cumulative impact exposure. The INDYCAR series protocol aligns with current international consensus statements and includes expert-informed multidimensional examination with reliable and valid tools composed of symptom report, neurocognitive evaluation, physical examination, vestibular assessment, and oculomotor functioning established each year during preseason baseline assessment and used in the trackside medical center in the event of a crash.^3,20,25^ Future studies exploring motorsport driver performance should use a longitudinal design across racing seasons combining multiple measurement modalities (eg, medical and neurological history, neurocognitive testing, advanced imaging, biomarkers, physical examination, accelerometer data, and estimated lifetime impact exposure) to understand potential neurobehavioral sequelae and long-term consequences associated with cumulative impact exposure.

A second limitation of this study is the reliance on postseason testing, limiting sensitivity to possible acute and subacute vestibular ocular dysfunction that normalized over time, consistent with other studies in athlete populations from other sports.^46,47,48^ Future studies should examine trends in oculomotor functioning in the acute, subacute, and long-term periods to determine whether professional motorsport drivers demonstrate comparable performance patterns. Third, consistent with limitations associated with a longitudinal design, this study is limited by driver attrition across racing seasons. We are currently conducting a companion study across 6 seasons that will address aspects of this limitation, because drivers not meeting current inclusion criteria (ie, participating in only 1 season within the current study window) will be included in follow-up analyses within the expanded time frame.

## Conclusions

The findings of this cohort study of INDYCAR series drivers reflect the relative stability of oculomotor performance despite exposure to the highest levels of cumulative impact conferred through multiple crashes over a 3-year period. To our knowledge, this study is the first to explore neurological outcomes of impact exposure in a professional motorsport cohort. Longitudinal studies across racing seasons that combine multiple measurement modalities (eg, neurocognitive testing, advanced imaging, biomarkers, and physical examination) are critical to characterize neurobehavioral sequelae and long-term consequences associated with the impact exposure unique to professional motorsport.

## Notes

Supplement 1. **Nonauthor Collaborators.** Johns Hopkins All Children’s Hospital/INDYCAR Research Collaboration

Supplement 2.
Data Sharing Statement
