# Supplementary material for: Longitudinal Outcomes of Cumulative Impact Exposure on Oculomotor Functioning in Professional Motorsport Drivers
Source: JAMA Netw Open. 2023 May 2;6(5):e2311086. doi: 10.1001/jamanetworkopen.2023.11086 (PMC10155066; doi:10.1001/jamanetworkopen.2023.11086)
Supplement: Supplement 1. — Nonauthor Collaborators. Johns Hopkins All Children’s Hospital/INDYCAR Research Collaboration [file jamanetwopen-e2311086-s001.pdf]

Supplemental Online Content: Nonauthor Collaborators
*First name, last name, and suffix (if applicable) are required and will appear in PubMed.

*Group Name(s): Johns Hopkins All Children's Hospital/INDYCAR Research Collaboration							
***First Name and Middle Initial(s)**	***Last Name**	***Suffix (eg, Jr, III)**	Academic Degrees	Institution	Location (city, state/province, country)	Role or Contribution, eg, chair, principal investigator	Group (if more than 1 Group listed in the byline) and/or Subgroup (eg, Steering Committee)
Geoff L.	Billows		MD	Indiana University School of Medicine	Indianapolis, IN	Medical Director for the INDYCAR Series and the Indianapolis Motor Speedway (2006-2022)	
